# Gravity-Induced Lower-Leg Swelling Can Be Ameliorated by Ingestion of α-Glucosyl Hesperidin Beverage

**DOI:** 10.3389/fphys.2021.670640

**Published:** 2021-06-23

**Authors:** Naoki Nishimura, Satoshi Iwase, Hiroko Takumi, Keiko Yamamoto

**Affiliations:** ^1^Department of Sport Sciences, Faculty of Sport Sciences, Nihon Fukushi University, Mihama, Japan; ^2^Department of Physiology, School of Medicine, Aichi Medical University, Nagakute, Japan; ^3^Institute of Health Sciences, Ezaki Glico Co., Ltd., Osaka, Japan; ^4^Okinawa Prefectural College of Nursing, Naha, Japan

**Keywords:** 4^*G*^-α-glucopyranosyl hesperidin, lower leg swelling, vascular permeability, gravity, skin surface temperature

## Abstract

The most likely cause of lower-leg swelling is prolonged sitting, which sometimes induces deep vein thrombosis, also known as, economy class syndrome. We aimed to clarify the influence of intake of 4^*G*^-α-glucopyranosyl hesperidin (G-Hsp) beverage on the lower-leg swelling caused by 6 h of sitting in six healthy women. All subjects ingested 100 mL of G-Hsp or Placebo beverages with 100 mL of mineral water after 10 min of rest in a chair. Subsequently, subjects were requested to sit in the chair in a relaxed position for 6 h with two breaks to walk for urination. Calf water content measured by impedance plethysmography, calf circumference, and calf skin temperature by infrared thermography were measured, along with assessment of calf swelling sensation on a visual analog scale. Increase in ankle % circumference was significantly less after the G-Hsp ingestion (101.8 ± 1.5%) than after placebo (103.3 ± 0.8%; *P* = 0.004). A significant difference was found between percent circumference after the G-Hsp and the placebo, that is, the calf swelling after the placebo was significantly larger (*P* = 0.043). A gradual increase in skin temperature at the lower limb was observed after G-Hsp ingestion, while there was no change after placebo. Gravity-induced calf and ankle swelling resulted by prolonged sitting can be ameliorated by oral ingestion of hesperidin-derived G-Hsp through production of nitric oxide. It might be helpful in preventing economy-class syndrome caused by enforced sitting for a long duration.

## Introduction

Foot, ankle, and lower-leg swelling refers to an accumulation of interstitial fluid in the lowest part of the body. It is a type of edema, which is an abnormal accumulation of interstitial fluid (Weber, 1932). Clinically, edema is significant swelling; the amount of interstitial fluid is determined by the balance of fluid homeostasis and the increased secretion of fluid into the interstitium, and the most likely cause of lower-leg swelling is prolonged sitting, which sometimes induces pulmonary embolism or deep vein thrombosis when blood clots leave and move to the lungs (Cruickshank et al., 1977; Lapostolle et al., 2001; Abunnaja et al., 2014). This lower-leg swelling is likely to occur in the lower limb in the daily lives of normal subjects (Yoneyama et al., 2007). In order to prevent this lower-leg swelling, several physical techniques have been implemented including dynamic action (Stick et al., 1992) and compression (Khoshgoftar et al., 2009) of the lower-leg muscles (the tibialis anterior, the soleus, and the gastrocnemius). However, oral ingestion of beverages has not been well examined yet.

**FIGURE 1:**
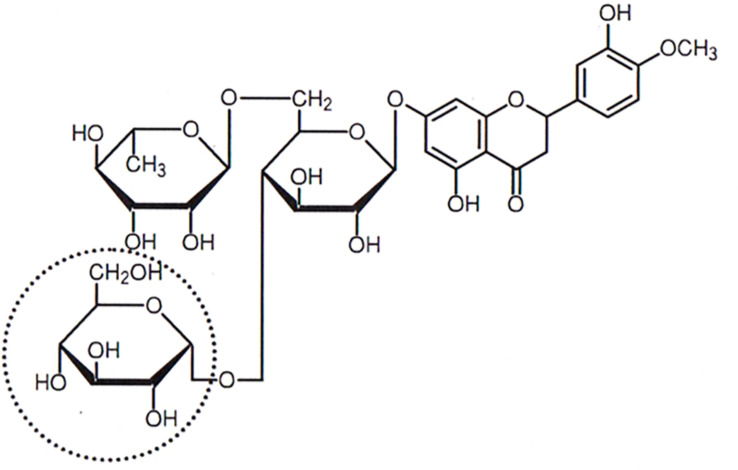
Structure of 4^*G*^-α-D-glucosylhesperidin.

The flavonoids including flavanones, flavone, and isoflavon are polyphenols present in the peel of such fruits as the mandarin orange (*Citrus unshiu* Marc.) in the form of glycosides. Hesperidin, one of the flavanone glycosides, is contained in the skin and flesh of some citrus fruits, and is considered to protect the fruit from ultraviolet rays, namely through vitamin P (Bentsáth et al., 1936). It has been shown to exert many biological activities, including reducing blood cholesterol levels and enhancing blood circulation (Garg et al., 2001). Hesperidin is one of the primary constituents of *Citrus unshiu* peel but is of limited use because of its low water solubility (Yamada et al., 2006). A hesperidin-derived compound, 4^*G*^-α-glucopyranosyl hesperidin (G-Hsp), by transglucosylation using cyclodextrin glucano-transferase (Figure 1) is much more soluble than conventional hesperidin (Kometani et al., 1994). Its absorbance rate to the human body is three times higher than that of hesperidin (Kometani et al., 2008). Moreover, it has been reported that G-Hsp can treat capillary fragility and permeability decrease (Garg et al., 2001; Liu et al., 2008), has an antioxidant effect (Hijiya and Miyake, 1991), and acts as an antiallergic (Galati et al., 1994) and antihypertensive (Galati et al., 1996). Thus, by enhancing the permeability of interstitial fluid into the blood vessel, G-Hsp might reduce lower-leg swelling.

We measured calf water content and calf circumference to examine the effect of G-Hsp ingestion on the prevention of the lower-leg swelling caused by prolonged sitting in healthy women. We hypothesized that the oral ingestion of a beverage containing dissolved G-Hsp would suppress water congestion at the lower leg in middle-aged women during prolonged sitting.

## Materials and Methods

### Subjects

Six healthy women served as the subjects. Their ages were 43 ± 2 years. They were all non-smokers and were taking no medications. They were given sufficient explanation and provided written informed consent. The protocol of the present study was approved by the institutional review board of Aichi Medical University. The study was conducted in accordance with the principles of the Declaration of Helsinki. Subjects were requested to abstain from caffeinated beverages, alcohol, citrus, and spices for at least 12 h before experimental sessions.

### Experimental Protocol

The subjects were requested to come to the laboratory at 10:00 h, at least 2 h after a light meal. All subjects were familiarized with the equipment and procedures before any experimental sessions. The experiments were carried out in an artificial climate chamber at an ambient temperature of 26°C and 50% relative humidity. The subjects change clothes a long-sleeved shirt and shorts pants after urination before start of experiments. After measuring of the circumference of the lower leg at the 1/2 and 1/4 sites between the patella and the malleolus of the subjects in a relaxed sitting position at the chair, an impedance electrode was applied halfway between the patella and the first toe. After 10 min of control reading, the subjects ingested 100 mL of test beverage with 100 mL of mineral water (total 200 mL) within a minute. The test beverage either contained dissolved G-Hsp or a placebo. The subjects were requested to come to the laboratory twice on other days, and the order of the beverages was selected at random (double blind study). After ingestion, they sat on a chair in a relaxed position for 6 h. At 2 h and 4 h after ingestion, they were requested to ingest 200 mL of mineral water, and they were allowed to walk for 30 s to the toilet next to the climatic chamber to urinate. During the 6 h of sitting, they were instructed to move as little as possible.

### Measurements

Calf water content was measured by impedance plethysmography using the bioelectrical impedance analysis (BIA) method (Kyle et al., 2004a,b) to analyze the body composition at the lower leg. This method measures the impedance between the two electrodes using an impedance plethysmograph (Nihon Koden AI-601G, Tokyo). Skin temperature at the lower limb was assessed by infrared thermography (Avio TVS-200EX, Tokyo Japan) every 30 min. The calf and ankle perimeters were measured every 30 min using a measuring tape. Subjective symptoms of calf swelling were rated every 30 min using a visual analog scale (VAS) between 0 and 100. Subjective symptoms of calf swelling were rated every 30 min using a VAS. This uses a 100 mm VAS, ranging from 0 (no swelling) to 100 (very severe swelling).

### Test Beverage

Each subject ingested 100 mL of a beverage containing 1,000 mg of G-Hsp with 100 mL of mineral water. The sweetness (sucrose) and acidic (citric acid) had been added to the beverage for taste the same to placebo beverage. A placebo beverage not containing G-Hsp was used for comparison. G-Hsp and placebo beverage were ingested on the other days in a random order.

### Statistics

All data are shown as the mean ± SE. Statistical significances between G-Hsp and placebo were calculated using two-way analysis of variance (ANOVA) followed by Greenhouse-Geisser or Huynh-Feldt multiple comparison tests. A *p*-value < 0.05 was considered significant. The VAS assessment was analyzed using the *t*-test.

## Results

### Changes in Calf Water Content During Sitting

**FIGURE 2:**
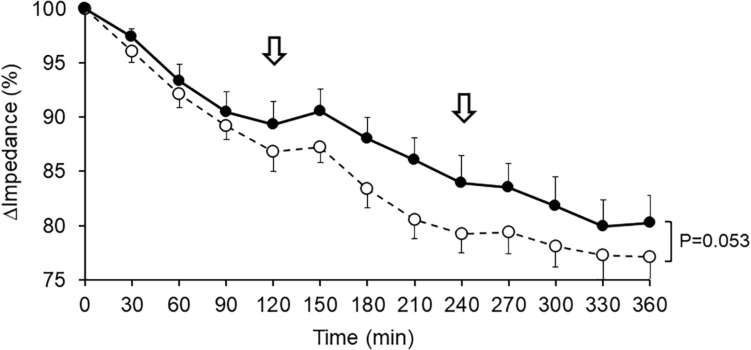
Changes in impedance of the lower leg after 1,000 mg of G-Hsp (•) and Placebo (∘) beverage ingestion. The arrow indicates the walking for urination, mean and SE, *n* = 6.

Since the impedance of biological tissue is a reciprocal of its water content, a decrease in impedance reflects an increase in water content. The calf impedance decreased gradually during sitting for 6 h after beverage ingestion. The percent difference from the control reading (taken as 100%), impedance tended to be smaller after G-Hsp ingestion than after the placebo (*P* = 0.053; Figure 2).

### Changes in Ankle Circumferences During Prolonged Sitting

**FIGURE 3:**
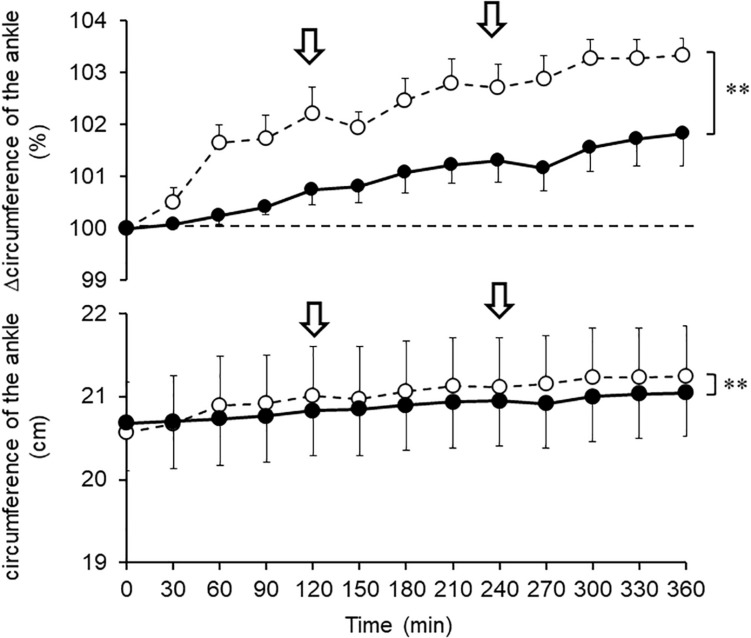
Changes in circumference of the ankle after 1,000 mg of G-Hsp (•) and Placebo (∘) beverage ingestion. The arrow indicates the walking for urination, mean and SE, *n* = 6, Asterisks indicate significant differences (^∗∗^*P* < 0.01).

The ankle circumference was increased with sitting time, and the absolute values of ankle circumference exhibited significant difference between G-Hsp and placebo (*P* = 0.002; Figure 3). Furthermore, there was a significant difference between the percent changes in circumference from the control reading after the G-Hsp beverage ingestion and after placebo. The increase in ankle percent circumference was significantly less after the G-Hsp ingestion (101.8 ± 1.5%) than after placebo (103.3 ± 0.8%; *P* = 0.004; Figure 3).

### Changes in Calf Circumferences During Prolonged Sitting

**FIGURE 4:**
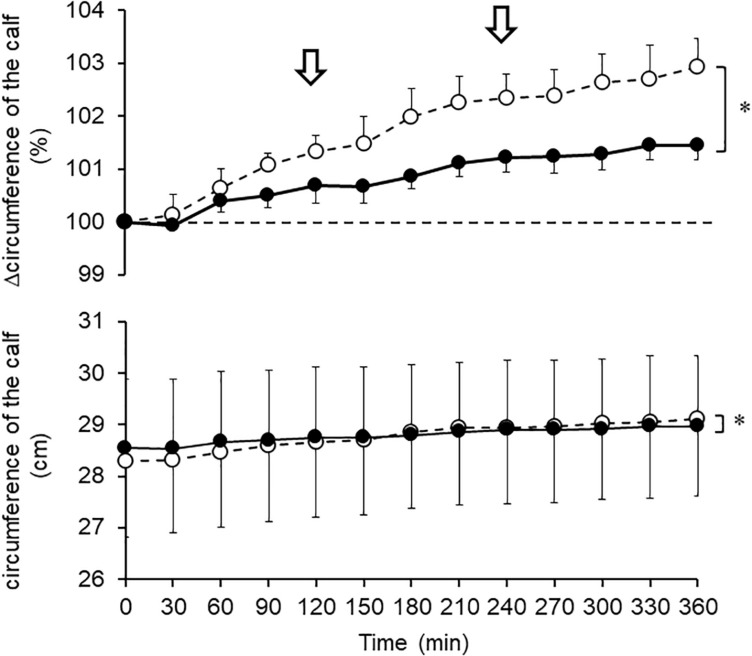
Changes in circumference of the calf after 1,000 mg of G-Hsp (•) and Placebo (∘) beverage ingestion. The arrow indicates the walking for urination, mean and SE, *n* = 6, Asterisks indicate significant differences (^∗^*P* < 0.05).

The calf circumference also increased with sitting time. A significant difference was found between percent circumference after the G-Hsp and the placebo, that is, the calf swelling after placebo was significantly larger (*P* = 0.043; Figure 4).

### Changes in Skin Surface Temperature During Prolonged Sitting

**FIGURE 5:**
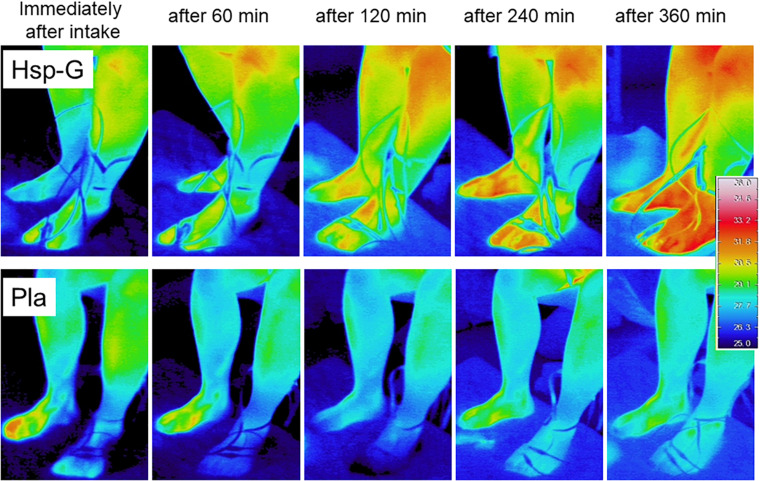
Comparison of skin temperature in the lower leg after 1,000 mg of G-Hsp (*upper panel*) and Placebo (lower panel) beverage ingestion in a subject.

A typical case of the skin surface temperature change shown by the infrared thermography is illustrated in Figure 5. In the lower-leg area, a gradual increase in the skin surface temperature was observed up to 6 h after G-Hsp ingestion. In contrast, a gradual decrease in the skin surface temperature was seen after placebo ingestion.

### Changes in Subjective Symptoms of Swelling During Prolonged Sitting

**FIGURE 6:**
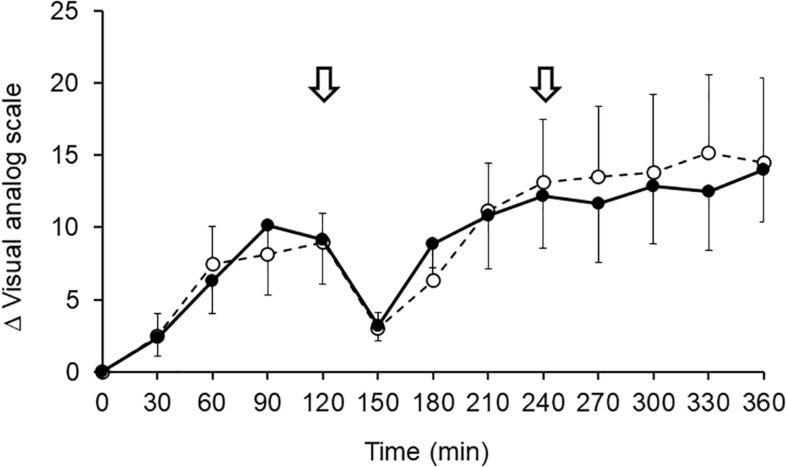
Changes in subjective symptoms of calf swelling using VAS scale after 1,000 mg of G-Hsp (•) and Placebo (∘) beverage ingestion. The arrow indicates the walking for urination, mean and SE, *n* = 6.

The VAS score for subjective symptoms of calf swelling is illustrated in Figure 6. The VAS score for subjective symptoms over time tended to be reduced by G-Hsp ingestion. However, the difference from the placebo was not significant.

## Discussion

The present study confirmed that the ankle swelling caused by prolonged sitting was ameliorated by the ingestion of a hesperidin derived compound, G-Hsp. This means that the ankle swelling was significantly larger after placebo ingestion.

The main findings of the present study were as follows: (1) the increase in calf water content induced by prolonged sitting as measured by bioimpedance analysis was suppressed by G-Hsp ingestion (Figure 2), (2) increases in the calf and the ankle circumference and subjective symptoms of swelling were suppressed by G-Hsp ingestion (Figures 3, 4, 6) and, (3) increases in skin temperature were facilitated by G-Hsp ingestion (Figure 5).

Symptoms of calf swelling are observed during and after prolonged standing or sitting, especially in women, and especially during pregnancy (Yoneyama et al., 2007). Venous clotting, especially pulmonary embolism or deep vein thrombosis, also known as, economy class syndrome, is one of the serious problems associated with prolonged sitting (Cruickshank et al., 1977; Lapostolle et al., 2001; Abunnaja et al., 2014), and dehydration caused by hypovolemia also contributes to this syndrome (Eklof et al., 1996). Hamada et al. (2002) reported that ionized beverage ingestion significantly ameliorates the condition. However, there has been no report on what kinds of supplement can facilitate the reduction of calf swelling.

4^*G*^-α-glucopyranosyl hesperidin is hydrolyzed into hesperidin by intestinal mucosal α-glucosidases, followed by its hydrolysis into aglycone hesperetin by β-glucosidase found in cytoplasm, and its absorption into the human body (Ohtsuki et al., 2002, 2003; Nielsen et al., 2006). It has been reported that G-Hsp is absorbed more efficiently and functions more effectively than hesperidin (Yamada et al., 2006). Various physiological functions of G-Hsp have been reported including serum lipid improvement (Miwa et al., 2005), bone metabolism improvement (Chiba et al., 2013), blood pressure decrease (Yamamoto et al., 2008), and reduced inflammation (Kometani et al., 2008).

In the present study, the calf and ankle swelling caused by prolonged sitting was possibly contributed to by femoral compression that reduced venous return and the effect of hydrostatic congestion in the lower extremities. This increased venous pressure can induce blood plasma to leak out of the vessels, eventually resulting in water retention in the interstitium. This calf and ankle swelling and subjective symptoms of swelling were ameliorated by ingestion of G-Hsp contained beverage.

The skin temperature was increased at 6 h after the G-Hsp ingestion, reflecting improved peripheral circulation through G-Hsp intake. A recent study on the suppression of tympanic and skin temperature decrease by exposure to cold confirmed the vasodilative effect and heat production caused by a G-Hsp ingestion, as well as a warmer sensation, particularly in women with high sensitivity to cold in the lower legs (Takumi et al., 2010). They administrated oral G-Hsp to the women for 4 weeks, and the subjects’ discomfort related to blood circulation and autonomic nervous system was ameliorated. In addition to this chronic administration study, the present acute administration study confirmed the effectiveness of G-Hsp on prevention of calf and ankle swelling as well as skin temperature maintenance.

The mechanism of blood circulatory improvement is considered to be dependent on nitric oxide production, inducing vascular dilatation (Takumi et al., 2011), since oral administration of G-Hsp for 3 weeks has been proved to promote nitric oxide production, reduced inflammation, and improved vascular endothelial cells (Rizza et al., 2011). In animal studies, administration of G-Hsp to spontaneous hypertensive rats were found to increase the bioavailability of smooth muscles, resulting in an increase in peripheral circulation and a decrease in systemic blood pressure (Yamamoto et al., 2008). Another study on the autonomic nervous system showed that G-Hsp administration to healthy women led to a significantly lower LF/HF ratio and higher HF component in heart rate variability, indicating vagal tone increase after ingestion of G-Hsp (Takumi et al., 2010).

In conclusion, gravity-induced calf and ankle swelling resulted by prolonged sitting can be ameliorated by oral ingestion of hesperidin-derived G-Hsp through production of nitric oxide. This may help people who complain of discomfort from lower-leg swelling and may provide a good measure for preventing economy-class syndrome.

## Data Availability Statement

The raw data supporting the conclusions of this article will be made available by the authors, without undue reservation.

## Ethics Statement

The studies involving human participants were reviewed and approved by the Aichi Medical University. The patients/participants provided their written informed consent to participate in this study.

## Author Contributions

NN and HT decided on the conception and design of the research. NN drafted the manuscript. NN, HT, and KY performed the experiments, analyzed the data, and interpreted the results of the experiments. All authors edited and revised the manuscript and read and approved the final manuscript.

## Conflict of Interest

HT was employed by the Ezaki Glico Co., Ltd., Osaka, Japan. The remaining authors declare that the research was conducted in the absence of any commercial or financial relationships that could be construed as a potential conflict of interest.
