# Corrigendum: Gravity-Induced Lower-Leg Swelling Can Be Ameliorated by Ingestion of α-Glucosyl Hesperidin Beverage

**DOI:** 10.3389/fphys.2021.739125

**Published:** 2021-09-09

**Authors:** Naoki Nishimura, Satoshi Iwase, Hiroko Takumi, Keiko Yamamoto

**Affiliations:** ^1^Department of Sport Sciences, Faculty of Sport Sciences, Nihon Fukushi University, Mihama, Japan; ^2^Department of Physiology, School of Medicine, Aichi Medical University, Nagakute, Japan; ^3^Institute of Health Sciences, Ezaki Glico Co., Ltd., Osaka, Japan; ^4^Okinawa Prefectural College of Nursing, Naha, Japan

**Keywords:** 4^*G*^-α-glucopyranosyl hesperidin, lower leg swelling, vascular permeability, gravity, skin surface temperature

In the original article, there was an error in the fourth paragraph of P2, MATERIALS AND METHODS, concerning the units of the Test Beverage that the subject consumed.

The authors have made the following correction:

**MATERIALS AND METHODS**

**Test Beverage**

**Error**

Each subject ingested 100 mL of a beverage containing 1,000 mL of G-Hsp with 100 mL of mineral water.

**Correct**

Each subject ingested 100 mL of a beverage containing 1,000 mg of G-Hsp with 100 mL of mineral water.

The authors apologize for this error and state that this does not change the scientific conclusions of the article in any way. The original article has been updated.

## Publisher's Note

All claims expressed in this article are solely those of the authors and do not necessarily represent those of their affiliated organizations, or those of the publisher, the editors and the reviewers. Any product that may be evaluated in this article, or claim that may be made by its manufacturer, is not guaranteed or endorsed by the publisher.

